# Retrospective study of changes in pharyngeal airway space and position of hyoid bone after mandibular setback surgery by cephalometric analysis

**DOI:** 10.1186/s40902-015-0039-8

**Published:** 2015-10-26

**Authors:** Hyun-Woo Cho, Il-Kyu Kim, Hyun-Young Cho, Ji-Hoon Seo, Dong-Hwan Lee, Seung-Hoon Park

**Affiliations:** 1 Department of Oral and Maxillofacial Surgery, College of Medicine, Inha University, #7-206, 3rd St. Shinheung-dong, Choong-gu, Incheon, 400-711 South Korea; 2 Department of Oral and Maxillofacial Surgery, International St. Mary’s Hospital, Catholic Kwandong University College of Medicine, Incheon, South Korea

**Keywords:** Pharyngeal airway space, Mandibular setback, Hyoid bone, Cephalometric

## Abstract

**Background:**

The posterior movement of mandible was known as the main cause of the changes in the pharyngeal airway space (PAS) and the postoperative obstructive sleep apnea (OSA). The purpose of this study was to know the changes of PAS and position of hyoid bone.

**Methods:**

Lateral cephalographies of 13 patients who had undergone sagittal split ramus osteotomy (SSRO) setback surgery were taken preoperatively (T1), postoperatively within 2 months (T2), and follow-up after 6 months or more (T3). On the basis of F-H plane, diameters of nasopharynx, oropharynx, and hypopharynx were measured. The movements of the soft palate, tongue, and hyoid bone were also measured.

**Results:**

The amount of mandible setback was 7.5 ± 3.8 mm. In the measurements of PAS, there was a statistically significant decrease of 2.8 ± 2.5 mm in nasopharynx (*P* < 0.01), and 1.7 ± 2.4 mm in oropharynx (*P* < 0.01) were observed after surgery. The hypopharynx decreased 1.0 ± 2.1 mm after surgery and continuously decreased 1.0 ± 2.8 mm at follow-up. The changes in hyoid bone position showed the posterior movement only after surgery and posteroinferior movement at follow-up.

**Conclusions:**

The PAS such as nasopharynx, oropharynx, and hypopharynx showed relatively high correlation with the amount of mandibular setback. The change of resistance in upper airway may be important for the prevention of OSA after mandibular setback surgery.

## Background

Airway management is an inseparable relation with surgery in maxillofacial region. During the postoperative period, airway management of patient is directly related to the vitality. In particular, at long-term follow-up, the narrowing of airway caused by surgery in the mandible or maxilla can make a serious impact on respiration in night time [1]. The respiratory disorders in night time such as obstructive sleep apnea (OSA) or snoring are known to affect the cardiovascular diseases which consequently affect mortality of patients [2, 3].

Sagittal split ramus osteotomy (SSRO) is recently the common surgery for the patient who has skeletal class III malocclusion. SSRO can change the position of mandible which improves occlusion and facial profile. However, some patients who received SSRO surgery were reported to be diagnosed with OSA syndrome [4]. For this reason, many studies have been reported the effects of SSRO setback surgery to the pharyngeal airway space (PAS).

The distal segment of mandible at SSRO setback surgery includes mandibular symphysis, body, and teeth. In addition, the tongue and hyoid bone are directly connected to the distal segment of mandible by muscles such as genioglossus muscle, geniohyoid muscle, and mylohyoid muscle. The posterior movement of this skeletal and soft tissue component in the mandible produces the consequent narrowing of PAS.

Therefore, it is important to distinguish the predisposing factors before operation in order to decrease the postoperative complication such as snoring or OSA associated with the narrowing of PAS. The factors affecting OSA generally have been known as body mass index (BMI), neck circumference, cigarette consumption, nasal stuffiness, or age [5]. Additionally, the factors related to mandible setback surgery have been reported in many studies as well [6–10].

The aim of this study is to evaluate the changes in PAS and position of hyoid bone in lateral cephalography associated with SSRO setback surgery. Furthermore, compensative position changes and relapse after surgery were also analyzed through the long-term follow-up.

## Methods

The subjects of this study were 13 patients diagnosed with skeletal class III malocclusion and underwent SSRO setback surgery between 2003 and 2014 at the Department of Oral and Maxillofacial Surgery, College of Medicine, Inha University, Incheon. The mean age of the patients was 22.4 years (range 18–29) at this surgery, and the patient group consisted of seven males and six females.

In this period, a total 285 patients who received SSRO surgery were reviewed. Exclusion criteria included SSRO for mandibular advancement and SSRO with involvement of maxillary Le Fort I osteotomy, anterior segmental osteotomy, genioplasty surgery, and other craniofacial anomalies. It also included those patients who did not take follow-up X-ray or people who taken in the wrong position of head. Lateral cephalography was taken to evaluate the amount of mandibular setback, pharyngeal airway space, and the movement of the hyoid bone. The phases taking lateral cephalography were divided into three groups: preoperatively (T1), postoperatively within 2 months (T2), and follow-up after 6 months or more from the operation (T3). The mean term of follow-up was 13.3 months.

**Fig. 1:**
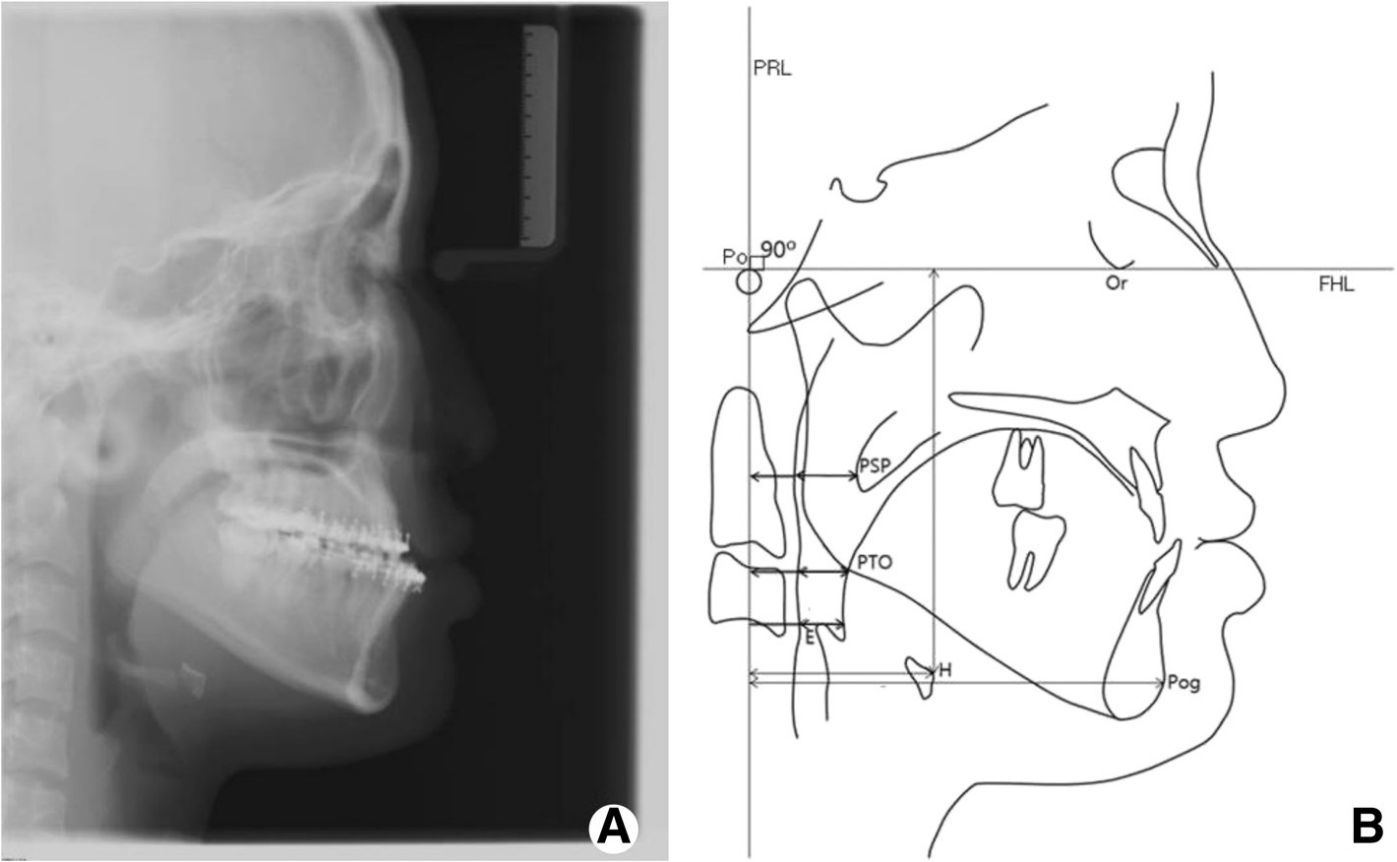
Reference landmarks and lines of the study. **a** Lateral cephalography. **b** Landmarks in analysis of pharyngeal airway space, movement of mandible, and position of hyoid bone. *Po* Porion, point located at the most superior point of the external auditory meatus; *Or* Orbitale, the lowest point in the inferior margin of the orbit; *Pog* Pogonion, the most anterior point on the contour of the symphysis; *PSP*, the most posterior point of the soft palate; *PTO*, point crossing mandibular inferior border in posterior area of the tongue; *E*, the most superior point of epiglottis; *H*, the most anterosuperior point of hyoid bone; *FHL*; *PRL*, line perpendicular to FHL passing Po

All tracing and analysis of the lateral cephalography were performed by one examiner in order to reduce the error. Two perpendicular planes were set up on the traced lateral cephalography as a basis for the measurement. F-H line (FHL) was drawn Porion (Po) to Orbitale (Or) as a horizontal reference line. Afterward, a line perpendicular to FHL passing Po (PRL) was drawn as a vertical reference line [11] (Fig. 1).

The distance of a line from the point of pogonion to PRL was measured to determine the amount of setback. The measurements of PAS were performed by following three linear variables: nasopharynx, oropharynx, and hypopharynx. The nasopharynx was measured as a distance of line which was the most posterior point of the soft palate (PSP) to posterior pharyngeal wall (PPW) and parallel with FHL. In the same way, the oropharynx was measured by point crossing mandibular inferior border in the posterior area of tongue (PTO) to PPW. The hypopharynx was measured by a distance of line between tongue base to PPW and passing through most superior point of epiglottis (E). Furthermore, similar linear variables were used in order to determine the change of tongue and soft palate. The soft palate was measured by distance from PSP to PRL and the position of tongue was measured by PTO to PRL. The position of hyoid bone was measured horizontally and vertically by distance of the most anterosuperior point of hyoid bone (H) to FHL and PRL.

### Statistics

All data was modified to actual size to compensate the magnification of images. Paired *t* tests were used to determine whether if there are any changes in each parameter have significance in their groups. Thus, Pearson’s correlation coefficient test was used so as to analyze the correlation between mandibular setback and PAS or position of hyoid bone.

## Results and discussion

**Fig. 2:**
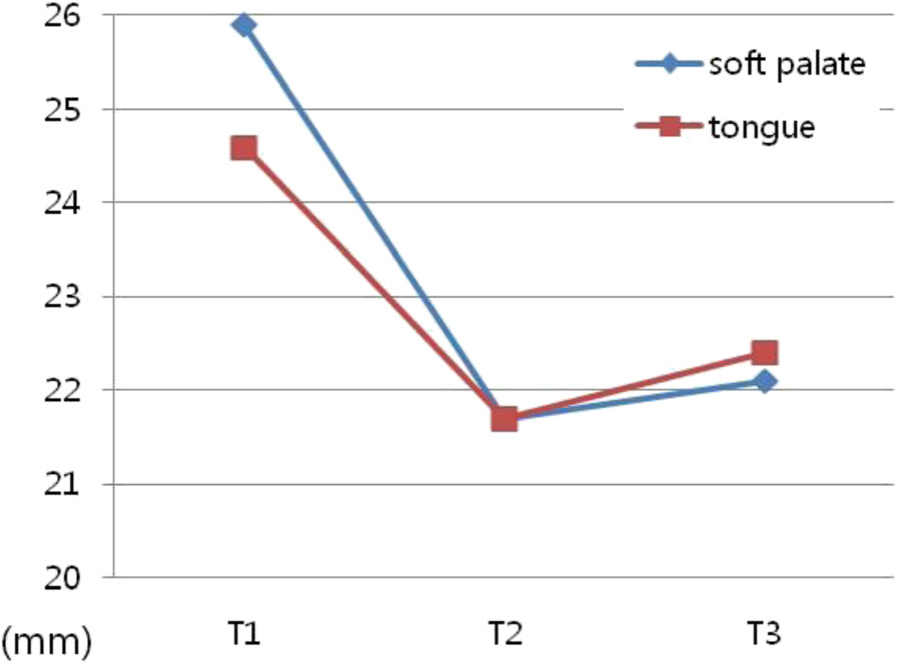
Trend of changes in soft tissue

**Fig. 3:**
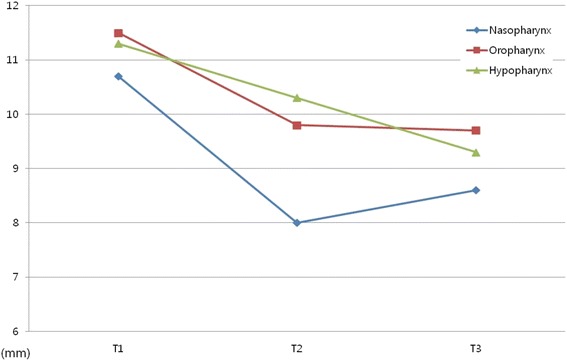
Trend of changes in pharyngeal airway space

**Fig. 4:**
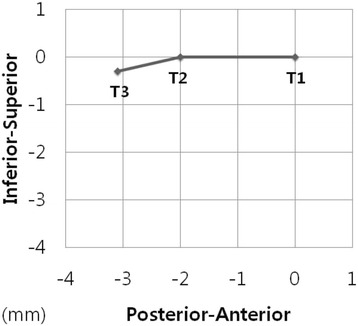
Changes in position of hyoid bone

The amount of mandible setback (PRL to Pog) was 7.5 ± 3.8 mm and postoperative relapse of mandible was 2.0 ± 2.8 mm protrusion. Tables 1 and 2 and Figs. 2 and 3 showed the trend of changes in PAS and soft tissue. In measurements of PAS, statistically significant decrease of 2.8 ± 2.5 mm in nasopharynx (*P* < 0.01) and 1.7 ± 2.4 mm in oropharynx (*P* < 0.05) was observed after surgery. The position of the soft palate and tongue moved posteriorly after surgery (Fig. 2). Compared with preoperative size of PAS, follow-up size of PAS (T3) showed statistically significant reduction. The changes in hyoid bone position showed the posterior movement only at the period between T1 and T2, and posteroinferior movement at the period between T2 and T3 (Fig. 4).

**Table 1:** Changes (mm) in pharyngeal airway space and soft tissue

	T1	T2	T3	T1, T2	T2, T3	T1, T3
Nasopharynx	10.7 ± 2.9	8.0 ± 3.0	8.6 ± 2.5	**	ns	**
Oropharynx	11.5 ± 3.9	9.8 ± 3.3	9.7 ± 3.1	**	ns	*
Hypopharynx	11.3 ± 2.5	10.3 ± 3.0	9.3 ± 2.5	ns	ns	**
Soft palate	25.9 ± 4.3	21.7 ± 3.9	22.1 ± 4.8	**	ns	**
Tongue	24.6 ± 4.6	21.7 ± 4.4	22.4 ± 5.7	**	ns	**
PRL to Pog	106.7 ± 8.8	98.0 ± 9.1	100.3 ± 9.9	**	*	**

*ns* not significant,

**P < 0.01, *P < 0.05

**Table 2:** Amount of changes (mm) in variables between T1 and T2, T2 and T3, T1 and T3

	T1-T2		T2-T3		T1-T3	
	Mean	SD	Mean	SD	Mean	SD
Nasopharynx	2.8	2.5	−0.6	2.6	2.1	1.9
Oropharynx	1.7	2.4	0.1	2.9	1.8	2.6
Hypopharynx	1.0	2.1	1.0	2.8	2.0	1.9
Soft palate	4.2	2.9	−0.5	3.3	3.8	3.7
Tongue	4.1	2.9	−0.7	3.3	2.2	2.4
PRL to H	2.0	2.3	1.0	3.6	3.1	4.3
FHL to H	0.0	5.4	−0.3	2.9	−0.3	3.7
PRL to Pog	7.5	3.8	−2.0	2.8	5.4	3.9

Pearson’s correlation coefficients toward the mandibular movement (PRL to Pog) were also measured (Table 3). According to this, the changes of variables are related to the movement of mandible. The PAS such as nasopharynx, oropharynx, and hypopharynx showed relatively high correlation with the amount of mandibular setback.

**Table 3:** Pearson’s correlation coefficient related to mandibular movement (PRL to Pog)

(PRL to Pog)	T1-T2	T2-T3	T1-T3
Nasopharynx	0.18	0.52	−0.23
Oropharynx	0.37	0.70	0.37
Hypopharynx	0.33	0.31	0.32
Soft palate	0.43	0.67	0.35
Tongue	0.45	0.74	0.57
PRL to H	0.57	0.08	0.55
FHL to H	−0.67	−0.01	−0.17

### Discussion

Accordingly, the soft tissues such as the lip, tongue, soft palate, and others will be affected at first when the mandible moves posteriorly. Marsan et al. [9] evaluated the changes of pharyngeal morphology of patients who had class III skeletal relationship and underwent mandibular setback surgery. The mean amount of setback was 4.3 mm after 1.5 years from surgery. Posterior movements of the soft palate and tongue were 3.1 and 3.2 mm. Saitoh [7] used the same reference plane to PSP and PTO to evaluate the soft tissue position after mandibular setback surgery. Evaluating periods were divided into before treatment, 3–6 months after SSRO, 2 or more years after SSRO. The mean amount of setback after 3–6 months and relapse from 3–6 months to 2 years or more were 8 and 0.4 mm. At 3-6 months after SSRO, the soft palate and tongue moved 6.0 and 9.2 mm posteriorly. The relapse rate of the soft palate and tongue from 3-6 months to 2 years or more was 56.7 and 71.7 %. In our study, the position of the soft palate and tongue posteriorly moved 4.2 and 4.1 mm at postoperative period when the amount of mandibular setback was 7.5 mm, and anteriorly moved 0.5 mm, 0.7 mm at follow-up when relapse of mandible was 2.0 mm (Tables 1 and 2 and Fig. 2). It showed positive correlation between mandibular movement and the soft tissue of upper airway (Table 3). The posterior repositioning of the soft palate may be caused by increased contact with the dorsum of the tongue as it moved back with the mandible [12].

Normally, the treatments of skeletal malocclusion are known to change the morphology of airway space in the oral region and maxillofacial surgery [13]. As the operations for mandibular prognathism were widely used, the retrospective study for the postoperative airway problem has performed by many researchers. Liukkonen et al. [14] evaluated the 22 patients who underwent bilateral vertical ramus osteotomies in 17 cases and bilateral sagittal ramus osteotomies in 5 cases. There was a statistically significant correlation between decrease in airway size and the mandibular setback at postoperative more than 1 year follow-up. They found out that the greater setback with posterior movement of mandible, the greater the reduction of airway size. Tselink and Pogrel [15] evaluated 14 patients who underwent bilateral sagittal split osteotomies to set back the mandible for correction of mandibular prognathism. At long-term follow-up, the mean reduction of the distance from the tongue base to the posterior pharyngeal wall was 4.77 mm from 18 mm which is the 28 % decrease of posteroanterior diameter compared with preoperative state.

Eggensperger et al. [6] reported PAS changes in 12 patients who underwent mandibular setback by SSRO with 12 years follow-up analysis of lateral cephalography. At long-term follow-up, lower pharyngeal airway was regained partly, but upper and middle pharyngeal airways showed continuous decrease.

In our study, nasopharynx showed 2.8 mm decrease after the surgery when the amount of mandibular setback was 7.5 mm. And, recovery of nasopharynx was 0.6 mm at follow-up which was 22 % compensation when the relapse of mandible was 2.0 mm. Oropharynx showed 1.7 mm decreased after the surgery and 0.1 mm continuous decrease at follow-up. And, the hypopharynx was decreased 1.0 mm after surgery and 1.0 mm at follow-up continuously (Tables 1 and 2 and Fig. 3). It showed positive correlation between the amount of mandibular setback and PAS (Table 3).

Gu et al. [16] studied a relation among hyoid position, PAS, and head posture after mandibular setback surgery. Postoperative movement of hyoid bone was posterior and downward direction. At the long-term follow-up, the hyoid bone moved anterior and superior, but it did not regain completely. In fact, there were other studies dealing with correlation between position of hyoid bone and change of pharyngeal airway space [6, 17, 18]. In our study, the changes in hyoid bone position showed the posterior movement only at the period after surgery, and posteroinferior movement at the period at follow-up (Tables 1 and 2 and Fig. 4).

Lateral cephalography has been used in the evaluation of PAS and hyoid bone position [6–9]. This cephalometric analysis has many advantages, such as a large body of research and study, low cost, and relatively low dose of radiation to patients. However, there are limitations that it can only show two-dimensional image in actual measurements of PAS and position of hyoid, lateral cephalography of skull. The lateral cephalography can give information such as a distance between two landmarks and sectional morphology of soft tissue. Therefore, computed tomography (CT) scan is necessary for the proper information of airway and position.

Degerliyurt et al. [19] studied a comparative CT evaluation of PAS on 47 patients who received bimaxillary surgery or mandibular setback surgery. CT scan was taken preoperatively within a week before surgery and postoperatively after at least 3 months. The study evaluated PAS in the anteroposterior and lateral dimensions. At the results in mandible setback surgery, the soft palate region showed 20 % decrease of anteroposterior dimension and 12 % decrease of lateral dimension. The base of tongue region showed 35 % decrease of anteroposterior dimension and 21 % decrease of lateral dimension. Kawamata et al. [20] reported that the reduction rates of anteroposterior and lateral dimension of PAS after 3 months from the surgery were 23.6 and 11.4 %. The diminished airway did not recover by either 6 months or 1 year after surgery. According to these CT studies, lateral dimension of PAS will be decreased along with anteroposterior dimension up to a certain amount. In our study, reduction rates of PAS was 19.7 % in nasopharynx, 15.7 % in oropharynx, and 17.7 % decrease in hypopharynx at T3 compared with T1.

Tomoko et al. [21] compared the PAS of patients who underwent orthodontic treatment alone and the PAS of patients who had orthodontic treatment and SSRO. At the initial visit, the PAS of surgery group showed the larger size which is statistically significant than that of orthodontic treatment group. Kim et al. [22] compared the groups between skeletal class II malocclusion and skeletal class III malocclusion which were classified by ANB difference. The oropharyngeal airway space of class III malocclusion group was greater. These results suggest that the patients with skeletal mandibular protrusion have wider pharyngeal airway than those with normal occlusion.

If upper airway is assumed to be the long cylinder, the resistance of air flow is proportional to the length of upper airway and inversely proportional to the fourth power of radius according to the Pouseilli’s law [23]. If the diameter of upper airway decreased 15 %, the resistance will increase approximately 1.92 times. The change of resistance in upper airway may be important for the prevention of OSA after mandibular setback surgery.

## Conclusions

The PAS such as nasopharynx, oropharynx, and hypopharynx showed relatively high correlation with the amount of mandibular setback. The change of resistance in upper airway may be important for the prevention of OSA after mandibular setback surgery.

## Consent

Written informed consent was obtained from the patients for publication of this Case report and any accompanying images. A copy of the written consent is available for review by the Editor-in-Chief of this journal.
